# Walsh Family Resilience Questionnaire—Polish Adaptation (WFRQ-PL)

**DOI:** 10.3390/ijerph19074197

**Published:** 2022-04-01

**Authors:** Natalia Nadrowska, Magdalena Błażek, Aleksandra Lewandowska-Walter, Wojciech Błażek, Agata Zdun-Ryżewska

**Affiliations:** 1 Department of Quality of Life Research, Faculty of Health Sciences, Medical University of Gdansk, 80-210 Gdansk, Poland; natalia.nadrowska@gumed.edu.pl (N.N.); azdun@gumed.edu.pl (A.Z.-R.); 2 Faculty of Social Sciences, Institute of Psychology, University of Gdansk, 80-309 Gdansk, Poland; aleksandra.lewandowska-walter@ug.edu.pl; 3 Faculty of Humanities and Social Sciences, Polish Naval Academy, 81-127 Gdynia, Poland; w.blazek@amw.gdynia.pl

**Keywords:** family resilience, family adaptation, developmental and unpredictable stress, WFRQ, FRAS

## Abstract

Family resilience is a construct based on interactive processes occurring in the family, enabling the family to effectively overcome everyday stressors, as well as developmental and unpredictable crises. By observing how the family deals with difficulties using family resilience processes, we are able to support both parents and protect children against the harmful effects of unfavourable conditions. The aim of our research was to carry out the procedure of adaptation to the Polish language and culture of the Walsh Family Resilience Questionnaire. In this study, 930 Poles participated (72.5% women), aged from 18 to 63 (M = 26.94, SD = 9.8). They filled in the questionnaire online. Confirmatory factor analysis confirmed the model with three factors: belief system, organisational processes, and communication processes. The model indicators were found to be well suited to the data: χ^2^/df = 1.12, RMSEA = 0.01, CFI = 0.99, TLI = 0.99, SRMR = 0.04. The reliability (Cronbach’s alpha) of the scales was also satisfactory (0.94 for the belief systems, 0.86 for the organisational processes, and 0.94 for the communication processes). Tool validation with FRAS-PL scales showed convergence. We named the Polish version of the WFRQ Questionnaire Kwestionariusz Prężności Rodzinnej Walsh (WFRQ-PL) and found it to be a good tool for assessing the processes of family resilience in our country.

## 1. Introduction

The processes of family resilience are based on family resources, and the family uses them during everyday stresses and crisis life events. These processes allow the family to withstand a stressful or crisis event, but also to grow stronger as a result of it. The beginnings of research on family resilience date back to the 1970s, when researchers focused on family interactions allowing a search to be made for resources inside and outside the family while enabling adaptation to unfavourable conditions [1]. Family resilience has redirected attention from the individual to the entire family. The family ceased to be only the context in which the behaviour of individual people was explained, and it became an object of interest in itself (the level of functioning of the family as a whole), with particular emphasis on the relations between its members. Family resilience is derived from research on mental resilience [2,3,4,5], research on family forces [6,7,8], and studies on stress and models of family resilience [9,10,11,12,13,14].

The approach to understanding the construct of family resilience has changed over time. Initially, family resilience was defined as a trait, dimension, or opportunity that helps the family survive and adapt to a difficult, crisis situation [12]. Despite drawing attention to patterns or relations occurring in the family, the aforementioned approach captures family resilience in the form of a set of features and processes that occur in the family, which are related to structural changes, as well as components related to the functioning of the family. Despite the fact that this approach draws attention to transformations resulting from the family life cycle and adaptive changes resulting from the impact of unfavourable events on the family, families are described in a binary way—as either resilient or non-resilient families. Resilience here is rather the result, not a process that indicates a positive solution to a crisis situation or the family coping with changes.

Then, family resilience was defined as the direction (path) in which families follow through successive stages of family life, in the face of unfavourable events, depending on numerous factors affecting the family (risk and protective) related to the circumstances of family life [15]. This approach takes into account the context of the stressful situation, the stage of family life, the relationship between protective and risk factors, a common family perspective; however, as Becvar (2013) points out, it ignores the diversity in family structures (e.g., single parents, reconstructed families) [16], cultural factors that affect the family [17], and the socioeconomic status of the family [18], as well as the interplay between the family and systems with which the family interacts [19,20,21].

Contemporary understanding of family resilience (Walsh, 2006) modifies and extends earlier approaches [22]. Family resilience in the approach created by Walsh (2006) refers to a process that develops over time in relation to a given difficult event and a specific phase of family life [22]. In addition, it takes into account the continuity of family life and the changes occurring in it. It is based on the developmental aspect of family resilience, which consists of the history of family life (previous difficult events), the accumulation of stress factors, intergenerational transmission, as well as recurring emotions. The processes of family resilience make it possible to endure difficult times, as well as to develop the potential and possibilities of both the whole family and each of its members. Overcoming everyday stresses, unfavourable events, and crisis situations strengthens and develops family resilience. In the discussed approach, difficulties (stressors) are perceived as challenges to the family.

According to Walsh (2006, 2013), each family shows resilience and, depending on many factors (phase of family life, history of family difficulties, type of event), uses various processes of family resilience that interact, cooperate, and complement each other [18,22]. It should be noted that in Walsh’s (2006) approach, the importance given to the meaning of resilience was transferred to processes constantly occurring in the family, such as belief systems, organisational patterns of family life, and communication processes. The approach of Walsh (2012) [21] draws attention to many contexts of family life (at the individual, family, community, and sociocultural levels) and takes into account their mutual interactions. It also takes into account numerous factors such as the socioeconomic status of the family, the culture in which the family lives and from which it originates, as well as the multiplicity and diversity of family structures. Using these processes, families strive to restore the balance in the system that has been disturbed as a result of the impact on the family: horizontal stress factors (normative, non-normative), vertical stressors (intergenerational heritage), and a significant accumulation of previous difficulties, as well as the reactivation of old but negative emotions as a result of current stressful events.

Walsh (2013) distinguishes three overarching processes of family resilience: the belief system, the organisational patterns of family life, and the process of communication and problem solving [18]. Each of the above-mentioned superior processes consists of three component processes. These processes, as Walsh (2006, 2016) points out, are dynamic, synergistic, and interactive, both within and between a given overarching process, which means that, e.g., communication and problem-solving processes will support the impact of flexibility and coherence processes [22,23].

The belief system influences the perception of stressful, difficult, and crisis situations, as well as reacting to them. It is created in society and passed down in families from generation to generation [24]. A shared belief system contributes to effective functioning and problem solving, as well as to an increase in resources and the strengthening of the family system [21,25]. In terms of the belief system, according to Walsh (2006), the model of family resilience processes consists of three subprocesses: giving meaning to adversity, positive outlook, transcendence, and spirituality [22]. The process of giving meaning to adversities in relation to the family system focuses on the relational aspect, i.e., the sense of understanding, resourcefulness, and meaningfulness expressed by all family members [26,27]. The process of having a positive attitude refers to the hope expressed by the family to improve the current and difficult situation, faith in overcoming adversities, taking initiative, focusing on potentials or strengths they have, putting efforts into aspects that can be changed, and accepting what cannot be changed [22,28]. Family members support one another in their efforts, motivate each other to take action, give encouragement and courage, and show support in their efforts to overcome unfavourable circumstances [29,30]. The process of transcendence and spirituality relates to several aspects such as faith, religious practices, contemplative practices, rituals, a superior system of values, membership of congregations and communities of people with a similar belief system, or expressed in a sense of union and harmony with the world and nature [31].

The organisational patterns of family life constitute the second overarching process of family resilience. The organisation of the family, depending on the phase of family life and the situation in which the family is located, looks different and depends on many factors influencing the family system [21,30]. This process is of great importance in the period of transformation and the transition between successive stages of family life, the accumulation of problems, family crises, and emerging unfavourable conditions for the life of the family [18,22]. The family can derive organisational patterns from relationships and values passed down from generation to generation, cultural and social norms, and expectations of society toward the family [22,28]. This process is composed of three subprocesses: flexibility, cohesion, and social and economic resources [22,32]. The process of flexibility refers to the family’s ability to reorganise the family system, to make changes in roles, tasks, or interaction patterns between members of the family system [18]. Mutual support, involvement in family life, cooperation toward a common goal while knowing the expectations of each family member and respecting their autonomy, and recognising boundaries and needs refer to the process of family cohesion [22]. The process of mobilising social and economic resources includes the ability of the family to ask for support from close or distant relatives, neighbours, and social institutions, ensuring financial security for the family and striving to meet basic living needs, as well as ensuring work–home balance [22].

The third overarching process of family resilience is communication and problem solving. Factors such as effective communication, actions focused on solving problems, and coping with stress, protect the family from the negative impact of risk factors [16]. Problem solving is facilitated by the relational model of communication, the partnership style of communication, and an active listening process [33]. The process of communication and problem solving consists of three subordinate processes: clarity of communication, open emotional expression, cooperation in problem solving [22]. Clarity of communication refers to the transmission of clear, authentic, unambiguous, and consistent messages by family members to one another. The process of open, emotional expression includes sharing feelings related to both positive and negative experiences, tolerance toward differences in feelings, and each family member’s reaction to events. One of the basic tasks of the cooperation process in solving problems is problem management, which includes recognition of the problem by all family members and focusing on actions aimed at solving the unfavourable situation or crisis the family experiences [22].

## 2. Materials and Methods

### 2.1. Aims of the Study

The main goal of our study was to carry out the procedure of adapting the Walsh Family Resilience Questionnaire [34] to the language and culture of the Polish population. We intended to assess the psychometric values of the tool, such as fitting the model to the data as well as the reliability of each of the scales. Our next goal was to test the validation of the Polish version of WFRQ with the Family Resilience Assessment Scale-PL [35].

### 2.2. Walsh Family Resilience Questionnaire

Walsh (2002) [26], based on her clinical work, experience, and a wide review of the literature, developed the Family Resilience Processes Model, which formed the groundwork for the Walsh Family Resilience Questionnaire [34], created by her. The WFRQ consists of three main scales: belief systems (13 items), organisational processes (9 items), and communication processes (10 items). By answering 32 questions, participants respond on a 5-point scale, from 1—rarely/never to 5—almost always, and rate to what extent individual statements are truthful about how their family is coping with crises and ongoing challenges. Below the quantitative family resilience items is a qualitative question about beliefs and practices that are especially helpful for the family to cope with difficult and stressful times.

Walsh (2016) points out that some processes of family resilience may be more important to a family than others in a difficult time, and we need to consider this in relation to the usefulness of the family’s resources and the situation that it has faced [23]. It follows that less important resources should not be treated as deficits in terms of family functioning in times of trouble. By extension, a higher score in the scope of a given family resilience process will indicate its wider and greater application than a process with a lower intensity.

Moreover, the answers given by family members on the scale can be used to conduct an in-depth interview, both in therapeutic practice and in qualitative research. The Walsh Family Resilience Questionnaire allows changes to be noticed over time in the processes of family resilience; it also reveals resources used by the family depending on the emerging challenges, adversities, and chronic stressors in their everyday life [34]. Another advantage of the questionnaire is that it can be used to assess the processes of family resilience before and after the intervention.

The data obtained in Walsh’s (2017) research using the questionnaire were not subjected to statistical analysis in order to obtain psychometric data, because, according to her, establishing a stable WFRQ factor structure may be something unattainable due to the interactive and recursive nature of family resilience processes [34]. In order to adapt the WFRQ, Walsh (2017) recommends taking into account the differences in culture, language, target population, and the nature of the crisis faced by a given group [34]. The Walsh Family Resilience Questionnaire has been adapted to the culture of Italy [36], Iran [37], China [38], Algeria, and Iraq [39], and people from a mid-South state of the US [40]. The questionnaire has so far been used, among others, in the study of autistic children [41], household disasters [42], bereavement [43], breast cancer patients [44], and families from various countries, such as Indonesia [45].

### 2.3. Procedure of Adaptation

Adapting the Walsh Family Resilience Questionnaire [34] to Polish conditions and culture required us to carry out several successive stages. We started working on the adaptation of the tool by obtaining permission to use the questionnaire from Froma Walsh. Then, three independent translators—psychologists fluent in English—translated the items from English into Polish. The obtained versions of the questionnaire were compared, and the common version was unified. The obtained version was used to test the pilot group (*N* = 15), who were asked to comment on incomprehensible concepts and to indicate unclear items. After minor linguistic corrections were made, the questionnaire was translated back into English by a psychologist, an English philologist. Next, the Polish version of the questionnaire’s translation, along with a reverse translation into English, was sent to Froma Walsh, who made a suggestion as to the meaning of individual items. We analysed the comments and agreed on the final version of the tool. Table 1 shows the WFRQ items translated into Polish.

Then, we started researching the target group. We adopted the following criteria for inclusion in the group: age over 18, living in Poland, Polish as the first language, and awareness of belonging to Polish culture and identifying with it. Participants filled in the questionnaire online. The link to the questionnaire was posted on the website of the Medical University of Gdańsk. We collected data for over a year and after collecting a sufficient number of responses, we proceeded to statistical analyses. We used confirmatory analysis with a DWLS estimator to check the suitability of the model and the Alpha Cronbach coefficient to analyse the internal reliability of the scales. The suitability of fitting the model to the data was determined by using the following indicators: root-mean-square error of approximation (RMSEA), comparative fit index (CFI), Tucker–Lewis index (TLI), and standardised root-mean-square residual (SRMR). In the article, we present the results of the suitability only for the model with 31 statements, due to the fact that the removal of item 22 did not significantly affect the measure of suitability and allowed us to maintain the items with the correct factor loadings (>0.03).

Our statistical analyses also included the reliability of the WFRQ scales, the correlation between WFRQ scales, as well as the assessment of the concurrent criterion validity of the WFRQ with the FRAS questionnaire. The Polish version of the Walsh Family Resilience Questionnaire we named Kwestionariusz Prężności Rodzinnej Walsh (WFRQ-PL). We performed statistical analyses with the use of R v. 4.0.5, RStudio v. 1.4.1717, and SPSS v. 27 software. The research was conducted with the consent of the Independent Bioethics Committee for Scientific Research at the Medical University of Gdansk.

### 2.4. Participants

Among a total of 930 participants, 674 (72.5%) were women and 256 (27.5%) men. They were aged from 18 to 63 (M = 26.94, SD = 9.8). There were 772 in early adulthood (aged 18 to 34) and 158 in middle adulthood (aged 35 to 65). The participants were native speakers of the Polish language who lived in Poland. The largest group were people living in cities with more than 500,000 inhabitants (*n* = 288, 31.0%) and rural residents (*n* = 254, 27.3%). Overall, 22.3% (*n* = 207) of people lived in cities with up to 100,000 inhabitants, and 19.5% in cities with 100,000 to 500,000 residents. Most of the participants in the group had secondary or technical education (*n* = 563, 60.5%), and higher education (*n* = 323, 34.7%), and the fewest were people with primary (*n* = 21, 3.3%) and vocational education (*n* = 13, 1.4%). In the questionnaire, just like Walsh, we left the open question below the questions rated on a 5-point scale. Nearly one-third (*n* = 301, 32.4%) of participants answered them. The characteristics of the group are presented in Table 2.

## 3. Results

### 3.1. Confirmatory Factor Analysis and Reliability of WFRQ-PL

The results of our research confirmed the original structure of the tool and statistical analyses and showed that the model with the three factors we tested was found to be a good fit for the data. The suitability indices were as follows: χ^2^/df = 1.12, RMSEA = 0.01, CFI = 0.99, TLI = 0.99, SRMR = 0.04. We kept 31 items with factor loadings higher than 0.3 and were statistically significant (*p* < 0.001). We had to remove item 22, as its factor values were too low (e = 0.25). The standardised factor loadings for the family belief system scale ranged from 0.51 to 0.86, for the family organisational processes from 0.35 to 0.87, and for the scale of communication and problem-solving processes from 0.69 to 0.85. Cronbach’s alpha for each of the scales was satisfactory and amounted to: 0.94 for the belief systems, 0.86 for the organisational processes, and 0.94 for the communication processes. Table 3 shows load values for each of the items.

### 3.2. Correlations between Scales of WFRQ-PL

Additionally, the correlations between the scores of the questionnaire were calculated. The correlations of participants between the scales of the questionnaire were found to be significant and strong. The results of these correlations are presented in Table 4.

### 3.3. Concurrent Validation

To carry out convergent validity, we used the Family Resilience Assessment Scale-PL scale (2017, 2021), previously adapted to Polish culture, which consists of six scales: family communication and problem solving, utilising social and economic resources, maintaining a positive outlook, family connectedness, family spirituality, and ability to make meaning of adversity. FRAS-PL is a good measure of criterion validity due to the fact that it is based on the same Model of Family Resilience Processes by Walsh (1996) as the WFRQ scale.

In this procedure, participants (N = 191), who were a subgroup in our study, aged 18 to 61 years (M = 28.25, SD = 10.29), 79.6% of whom were women, with mainly secondary education (60.7%) and higher (38.2), and residents of Poland completed two questionnaires online: WFRQ-PL and FRAS-PL.

In order to assess the convergent criterion validity of WFRQ-PL, we performed an analysis of the Pearson R correlation between WFRQ-PL and FRAS-PL scales. We obtained moderate and statistically significant correlations between WFRQ-PL and FRAS-PL scales. Only the family spirituality scale (FRAS-PL) did not correlate with any of the three WFRQ-PL scales. The results are presented in Table 5.

## 4. Discussion

The main goal of our study was to carry out the procedure of adapting the Walsh Family Resilience Questionnaire [34] to the language and culture of the Polish population. The importance of adapting the tool is underlined by the increasing amount of research in the field of resources, individual resilience, and family resilience. The assessment of family resilience should take into account a multitude of factors, including the nature of the stressor, the stage of family life, the scope of the stressor’s impact, reactions to the stressor of each family member, ways of dealing with everyday stressors and normative and non-normative crises by the family in the past and present, and the social, cultural and economic situation of the family [30]. Depending also on the social and economic situation of the family, and the cultural context, the processes of family resilience may take a different course. Considering the importance of the family environment and its specific situation, the phenomenon of resilience fits well with systemic family theories. The systemic view of family resilience allowed us to focus on patterns of adaptation within families and to observe children’s functioning and the quality of their bonds with those from the family system, as well as those a little further afield, from the community system [46]. The usefulness of such an approach is explained by the significant increase in research on the resilience of families in which children are brought up, starting from research on child functioning in immigrant families [47], and families with children growing under difficult conditions [4,5,48], to how families dealing with crises connected, for example, with war [49], or health problems [50]. How and how quickly the family copes with everyday stressors and recovers from various types of disruptions (resulting from stages of the family’s life as well as extraordinary situations) is an area of particular attention in the field of systems theory in the context of child functioning. Walsh’s theory [22], and the scale (WFRQ-PL) [34] we adapted, can be very useful in this context. Resilience is an important factor in the process of rebalancing, and the scale we proposed gives psychologists the opportunity to diagnose and design help for the entire family system in order to best support the child [51]. In contrast to the classical model of psychological help, i.e., focusing on deficits and problems, in this case, it is possible to broaden it to include the identification and promotion of adaptive processes for children and for the whole family (with delineation of pathways for the adaptive function in children and the family, combined with an understanding of mutual connections between both of them), and the identification of risks to positive adaptation and protective processes that might be used in the work with the family [52].

Until now, an adapted Family Resilience Assessment Scale-PL questionnaire [35,53]—has been used to assess family resilience in Poland. The FRAS questionnaire [24] was developed on the basis of the Walsh Family Resilience Model (2006) [22] and consists of six scales—namely, family communication and problem solving, utilising social and economic resources, maintaining a positive outlook, family connectedness, family spirituality, and ability to make meaning of adversity. The structure of the original FRAS questionnaire was confirmed in Poland [35]. Due to the complexity of the family resilience construct, the aim of our research was to adapt another tool—the Walsh Family Resilience Questionnaire—to the Polish language and culture, as well as to analyse its psychometric values.

The confirmatory factor analysis showed that the original three-factor structure of the Walsh Family Resilience Questionnaire is a good fit for the data collected and analysed by us. Due to its low load, we decided to delete item 22. Deleting the statement did not significantly affect the fit measures but allowed us to leave items about good factor loading. The Polish version of the WFRQ-PL questionnaire consists of 31 questions which are included in the three scales: belief system, organisational processes, and communication processes. Like Walsh (2017) [34], we left a qualitative question, about family-supporting practices in coping with crises. We also obtained statistically significant and strong correlations between the WFRQ-PL scales, which confirms Walsh’s theories about the interactivity of family resilience processes. The reliability for each of the scales also proved to be satisfactory, and we also confirmed the convergent criteria validity with the scales of the FRAS-PL questionnaire. The exception was the family spirituality scale, which did not correlate with any of the WFRQ-PL scales. We argue this by the fact that the spirituality scale of FRAS focuses mainly on religious practices, while the belief system scale of WFRQ focuses on the teleonomic aspects of spirituality and transcendence, based on making meaning to given events, understanding them, and overcoming crises through faith and hope. Furthermore, the overarching process of the belief system was included by Sixbey (2005) [24] in three separate scales in the FRAS questionnaire—maintaining a positive outlook, family spirituality, ability to make meaning of adversity—while in Walsh (2016) [23], these substructures are concentrated within the one belief system scale, which proves the different structure of both tools and emphasises the diversity of the complexity of understanding the processes of family resilience. The differences in the conceptualisation of the superior scales prove the structural differences of both tools and emphasise the diversity and complexity of understanding family resilience processes.

A three-factor solution similar to the Polish adaptation was obtained in the mid-South state of the US [40], China [38], Italy [36], and in research on bereaved families [43]. However, in the studies of participants from Iran [37], Iraq, and Algeria [39], a nine-factor model was achieved. Moreover, in the adaptation made to the Italian population and bereaved adults who had lost a family member, the general scale of family resilience was kept. What should be noted in bereaved families is that the largest contribution to the general factor is the belief system scale. The Chinese [38] and Italian [36] versions consist of 26 questions, while the 31- or 32-item versions were obtained in other countries by adapting the Walsh Family Resilience Questionnaire as in Poland or Iran [37].

We can indicate some theoretical and practical implications for the questionnaire of WFRQ adapted to the Polish language and culture. The adaptation of the questionnaire for the assessment of family resilience processes contributes to family psychology, especially in the area of family resources and strength. Polish culture is characterised by the high position of the family as a value and the belief that there is a need to solve problems within the family [54]. By adapting the WFRQ, we gained an additional tool that measures three overarching components of the original Walsh model: belief system, organisational processes, and communication processes. This will allow for the design of further studies that will contribute to the development of the family resilience construct in the various fields of family functioning in times of daily stresses and crisis. The use of the questionnaire allows the assessment of what resources the family has, in addition to emphasising their importance for future opportunities and the development of family potential. The WFRQ-PL questionnaire can be used in two ways. First, it can be applied to examine each family member individually to see if family members perceive family resources for coping with stress in the same or different ways. Secondly, as Lewandowska-Walter and Błażek (2018) mention in their research on families, we recommend using the questionnaire to study the entire family system, i.e., when family members complete one questionnaire together [55]. Then, we can also observe the communication, negotiation, and emerging tensions and conflicts between them. The study of family members with the WFRQ-PL questionnaire is the basis for an in-depth qualitative interview on the resources used by the family. The use of the method in therapeutic practice makes family members aware and sensitive to the positive potential of a family in a crisis situation. Using the questionnaire several times during the therapeutic process allows an understanding of how the processes of family resilience change over time, which will allow the therapist to maintain or introduce modified techniques for working with the family. On the other hand, it will allow the family to make changes in their methods of coping with difficulties. By contributing to the development of the potential of positive coping through the processes of family resilience manifested by adult family members, i.e., parents or guardians, children acquire the most adaptive patterns of functioning in a stressful situation. First, in the therapeutic process, by observing how the family copes with difficulties, we are able to support both parents and protect children against the harmful effects of unfavourable conditions.

As in most cases, our study also has limitations. One is that the majority of the study group are participants in early adulthood, mostly women. We did not conduct a qualitative interview with the participants and did not divide the group in terms of experienced crises. We focused on the study of family resilience in a representative group of Polish families. Another limitation is that the WFRQ-PL questionnaire was completed only by a family representative, possibly by two members of the family, and not by the entire family. Research on the processes of family resilience and the use of the WFRQ questionnaire are in the initial phase and will be continued.

In our future research, however, we intend to introduce a qualitative interview to expand the information obtained in the WFRQ questionnaire and increase the group of men and people over 35 to balance the group and be able to start the standardisation process. We are going to focus on specific crises and thus differentiate the family resilience of the participants. Conducting the research on a representative group of the Polish population will allow us to compare the achieved results to the results obtained in specific life crises. We also intend to use a questionnaire to test at least 2–3 members from the same family and not only one representative of a given family.

## 5. Conclusions

1. The adaptation of the WFRQ to the Polish language and culture was successful. The questionnaire consists of 31 items and 1 quality question;
2. The questionnaire adapted to Polish conditions allows for comparisons between countries in terms of the processes of family resilience;
3. The Walsh Family Resilience Questionnaire is a tool for adults for assessing family resilience processes such as belief systems, organisational processes, and communication processes in times of daily stress and crisis situations in life;
4. WFRQ-PL research requires continuation, increasing the number of men, people over 35, and study on families in crises;
5. WFRQ-PL might be useful in scientific research and practical work with families of different structures at different stages of family life, experiencing a variety of crises, including everyday stressors;
6. Therapists using the WFRQ-PL questionnaire to observe how the family deals with difficulties by using resilience processes will be able to support both parents and protect children against the harmful effects of unfavourable conditions.

## Figures and Tables

**Table 1:** Polish translation of Walsh Family Resilience Questionnaire items.

Item	English Version	Polish Translation
1.	Our family faces difficulties together as a team, rather than individually.	Nasza rodzina radzi sobie z trudnościami wspólnie (jak drużyna), a nie każdy z osobna
2.	We view distress with our situation as common, understandable.	Stres, który pojawia się w życiu naszej rodziny, spostrzegamy jako coś zwykłego i zrozumiałego
3.	We approach a crisis as a challenge we can manage and master with shared efforts.	Kryzys jest dla nas wyzwaniem, z którym radzimy sobie wspólnym wysiłkiem
4	We try to make sense of stressful situation and focus on our options.	Staramy się nadawać sens stresującym sytuacjom i skupić się na możliwych rozwiązaniach
5.	We keep hopeful and confident that we will overcome difficulties.	Mamy nadzieję i wierzymy, że przezwyciężymy wszystkie trudności
6.	We encourage each other and build on our strengths.	Wspieramy się nawzajem i wykorzystujemy nasze mocne strony
7.	We seize opportunities, take action, and persist in our efforts.	Wykorzystujemy możliwości, podejmujemy działania i jesteśmy wytrwali w naszych wysiłkach
8.	We focus on possibilities and try to accept what we cannot change.	Skupiamy się na możliwościach i próbujemy zaakceptować rzeczy, których nie można zmienić
9.	We share important values and life purpose that help us rise above difficulties.	Nasze wspólne wartości i cele życiowe pomagają nam przezwyciężyć trudności
10.	We draw on spiritual resources (religious or non-religious) to help us cope well.	Lepiej radzimy sobie z trudnościami dzięki wartościom duchowym (religijnym i niereligijnym)
11.	Our challenges inspire creativity, more meaningful priorities, and stronger bonds.	Wyzwania pobudzają naszą kreatywność, przewartościowują priorytety i budują silniejsze więzi między nami
12.	Our hardship has increased our compassion and desire to help others.	Doświadczane trudności rozwinęły w nas współczucie i pragnienie pomagania innym
13.	We believe we can learn and become stronger from our challenges.	Trudności, których doświadczamy, stanowią dla nas lekcje i umacniają nas
14.	We are flexible in adapting to new challenges	Elastycznie przystosowujemy się do nowych wyzwań
15.	We provide stability and reliability to buffer stresses for family members.	Możliwość zyskania oparcia w rodzinie i polegania na sobie nawzajem chroni nas przed doświadczanym stresem
16.	Strong leadership by parents/caregivers provides warm nurturing, guidance, and security.	Silne przywództwo rodziców/opiekunów zapewnia czułą opiekę, wsparcie i poczucie bezpieczeństwa
17.	We can count on family members to help each other in difficulty.	Możemy liczyć na to, że członkowie naszej rodziny będą pomagali sobie w trudnych sytuacjach
18.	Our family respects our individual needs and differences.	Szanujemy pojawiające się między nami różnice i indywidualne potrzeby każdego członka rodziny
19.	In our immediate and extended family, we have positive role models and mentors.	W naszej bliższej i dalszej rodzinie możemy odnaleźć osoby, które są dla nas wzorcami do naśladowania i mentorami
20.	We can rely on the support of friends and our community.	Możemy liczyć na wsparcie przyjaciół i osób z naszego otoczenia
21.	We have economic security to be able to get through hard times.	Posiadamy oszczędności, które pozwolą nam przetrwać trudny czas w życiu
22.	We can access community resources to help our family through difficult times.	Możemy skorzystać ze wsparcia instytucji społecznych, aby przetrwać trudny czas
23.	We try to clarify information about our stressful situation and our options.	Staramy się znaleźć przyczynę i rozwiązanie w trudnej sytuacji w której się znaleźliśmy
24.	In our family, we are clear and consistent in what we say and do.	Działamy zgodnie z podjętymi ustaleniami
25.	We can express our opinions and be truthful with each other.	W naszej rodzinie możemy otwarcie i szczerze wyrażać swoje opinie
26.	We can share difficult negative feelings (e.g., sadness, anger, fears).	W naszej rodzinie możemy dzielić się z innymi naszymi trudnymi uczuciami (takimi jak smutek, złość, strach)
27.	We show each other understanding and avoid blame.	Okazujemy sobie zrozumienie i unikamy oskarżeń
28.	We can share positive feelings, appreciation, humor, and fun and find relief from difficulties.	Pozytywne emocje, wspólna zabawa, okazywanie wdzięczności i poczucie humoru przynoszą nam ulgę w trudnym czasie
29.	We collaborate in discussing and making decisions, and we handle disagreements fairly.	Wspólnie dyskutujemy, współpracujemy przy podejmowaniu decyzji i w sposób uczciwy rozwiązujemy konflikty
30.	We focus on our goals and take steps to reach them.	Koncentrujemy się na naszych celach i podejmujemy kroki, aby je osiągnąć
31.	We celebrate successes and learn from mistakes.	Wspólnie cieszymy się z sukcesów oraz uczymy się na błędach
32.	We plan and prepare for the future and try to prevent crises.	Robimy plany na przyszłość i staramy się zapobiec możliwym kryzysom

**Table 2:** Characteristic of participants (*N* = 930).

Gender, % (*n*)	
Female	72.5 (674)
Male	27.5 (256)
Age, M ± SD	26.94 ± 9.82
Range of age	18–63
>35, % (*n*)	83% (772)
35–63, % (*n*)	17% (158)
Level of education, % (*n*)	
Primary	3.3 (31)
Vocational	1.4 (13)
Secondary	60.5 (563)
Higher	34.7 (323)
Place of residence, % (*n*)	
The country	27.3 (254)
City up to 100,000 residents	22.3 (207)
City from 100,000 up to 500,000 residents	19.5 (181)
City over 500,000 residents	31.0 (288)

**Table 3:** Factor loadings for WFRQ-PL.

	Estimate	Std. Estimate	*p*
Belief Systems			
1.	1.000	0.789	0.000
2.	0.547	0.512	0.000
3.	0.992	0.813	0.000
4	0.910	0.780	0.000
5.	0.880	0.790	0.000
6.	1.045	0.856	0.000
7.	0.937	0.848	0.000
8.	0.806	0.749	0.000
9.	1.019	0.836	0.000
10.	0.649	0.442	0.000
11.	0.916	0.767	0.000
12.	0.797	0.650	0.000
13.	0.826	0.738	0.000
Organisational Processes			
14.	1.000	0.718	0.000
15.	1.419	0.873	0.000
16.	1.155	0.690	0.000
17.	1.049	0.756	0.000
18.	1.193	0.800	0.000
19.	0.993	0.594	0.000
20.	0.541	0.404	0.000
21.	0.529	0.352	0.000
Communication Processes			
23.	1.000	0.733	0.000
24.	0.828	0.690	0.000
25.	1.127	0.749	0.000
26.	1.282	0.819	0.000
27.	1.196	0.802	0.000
28.	1.059	0.761	0.000
29.	1.257	0.846	0.000
30.	1.080	0.819	0.000
31.	0.993	0.788	0.000
32.	0.990	0.687	0.000

**Table 4:** Correlations between scales of WFRQ-PL.

	Organisational Processes	Communication Processes
Belief Systems	0.821 ***	0.871 ***
Communication Processes	0.841 ***	1

*** *p* < 0.001.

**Table 5:** Correlations between the 31-item Walsh Family Resilience Questionnaire-Poland Version (WFRQ-PL) factors and validation instruments—FRAS-PL.

	FRAS-PL	FCPS	USER	MPO	FC	FS	AMMA
WFRQ-PL							
Belief Systems		0.455 ***	0.329 ***	0.416 ***	0.369 ***	0.142	0.254 ***
Organisational Processes		0.424 ***	0.435 ***	0.378 ***	0.357 ***	0.121	0.168 *
Communication Processes		0.567 ***	0.410 ***	0.484 ***	0.459 ***	0.025	0.285 ***

FCPS—family communication and problem solving, USER—utilising social and economic resources, MPO—maintaining a positive outlook, FC—family connectedness, FS—family spirituality, AMMA—ability to make meaning of adversity. * *p* < 0.05; *** *p* < 0.001.

## References

[1] McCubbin H.I., McCubbin M.A., Grasso A.J., Epstein I. (1992). Research Utilization in the social work practice of family treatment. Research Utilization in the Social Sciences: Innovations for Practise and Administration.

[2] Luthar S.S. (1991). Vulnerability and Resilience: A Study of High-Risk Adolescents. Child Dev..

[3] Masten A.S. (2001). Ordinary Magic: Resilience Processes in Development. Am. Psychol..

[4] Garmezy N., Stevenson J. (1985). Stress-Resistant Children: The Search for Protective Factors. Recent Research in Developmental Psychopatology.

[5] Werner E.E. (1995). Resilience in Development. Curr. Dir. Psychol. Sci..

[6] Olson D.H. (2000). Circumplex Model of Marital and Family Systems. J. Fam. Ther..

[7] Stinnett N., DeFrain J. (1985). Secrets of Strong Families.

[8] Woodhouse C.G. (1930). A Study of 250 Successful Families. Soc. Forces.

[9] Hill R. (1949). Families under Stress: Adjustment to the Crises of War Separation & Reunion.

[10] Lavee Y., McCubbin H.I., Patterson J.M. (1985). The Double ABCX Model of Family Stress and Adaptation: An Empirical Test by Analysis of Structural Equations with Latent Variables. J. Marriage Fam..

[11] McCubbin H.I., Patterson J.M. (1983). The Family Stress Process: The Double ABCX Model of Adjustment and Adaptation. Marriage Fam. Rev..

[12] McCubbin M.A., McCubbin H.I., Figley C.R. (1989). Theoretical orientations to family stress and coping. Treating Stress in Families.

[13] McCubbin M.A., McCubbin H.I., Danielson C.B., Hamel-Bissell B., Winsted-Fry P. (1993). Families coping with illness: The resiliency model of family stress, adjustment and adaptation. Families, Health and Illness: Perspectives on Coping and Intervention.

[14] McCubbin M.A., McCubbin H.I., McCubbin H.I., Thompson A.I., McCubbin M.A. (1996). Resiliency in families: A conceptual model of family adjustment in response to stress and crises. Family Assessment: Resiliency, Coping and Adaptation—Inventories for Research and Practice.

[15] Hawley D.R., DeHaan L. (1996). Toward a Definition of Family Resilience: Integrating Life-Span and Family Perspectives. Fam. Process.

[16] Becvar D.S., Becvar D.S. (2013). Facilitating family resilience in clinical practice. Handbook of Family Resilience.

[17] McGoldrick M., Giordano J., Pearce J.K. (1996). Ethnicity and Family Therapy.

[18] Walsh F., Becvar D.S. (2013). Community-based practice applications of a family resilience framework. Handbook of Family Resilience.

[19] Bronfenbrenner U. (1979). The Ecology of Human Development.

[20] McGoldrick M., Carter B., Walsh F. (2015). The family life cycle. Normal Family Processes: Growing Diversity and Complexity.

[21] Walsh F., Walsh F. (2012). The “new normal”: Diversity and complexity in 21st century families. Normal Family Processes: Growing Diversity and Complexity.

[22] Walsh F. (2006). Strengthening Family Resilience.

[23] Walsh F. (2016). Family Resilience: A Developmental Systems Framework. Eur. J. Dev. Psychol..

[24] Sixbey M.T. (2005). Development of the Family Resilience Assessment Scale to Identify Family Resilience Constructs. Ph.D. Thesis.

[25] Walsh F., Walsh F. (2012). Clinical views of family normality, health, and dysfunction. Normal Family Processes: Growing Diversity and Complexity.

[26] Walsh F. (2002). A Family Resilience Framework: Innovative Practice Applications. Fam. Relat..

[27] Walsh F., Borden W. (2010). A family resilience framework for clinical practice: Integrating developmental theory and systemic perspectives. Reshaping Theory in Contemporary Social Work: Toward a Critical Pluralism in Clinical Practice.

[28] Walsh F. (1996). The Concept of Family Resilience: Crisis and Challenge. Fam. Process.

[29] Walsh F. (2008). Using theory to support a family resilience framework in practice. Soc. Work.

[30] Walsh F. (2003). Family Resilience: A Framework for Clinical Practice. Fam. Process.

[31] Walsh F. (2010). Spiritual Diversity: Multifaith Perspectives in Family Therapy. Fam. Process.

[32] Walsh F. (2016). Applying a Family Resilience Framework in Training, Practice, and Research: Mastering the Art of the Possible. Fam. Process.

[33] Rostowska T. (2008). Małżeństwo, Rodzina, Praca a Jakość Życia.

[34] Walsh F. (2017). Strengthening Family Resilience.

[35] Nadrowska N., Błażek M., Lewandowska-Walter A. (2021). Polish Adaptation of the Family Resilience Assessment Scale (FRAS). Community Ment. Health J..

[36] Rocchi S., Ghidelli C., Burro R., Vitacca M., Scalvini S., Della Vedova A.M., Roselli G., Ramponi J.P., Bertolotti G. (2017). The Walsh Family Resilience Questionnaire: The Italian Version. Neuropsychiatr. Dis. Treat..

[37] Haji D., Karaminia M., Salimi R., Ahmadi Tahour S.H. (2018). Translation and Validation of the “Walsh Family Resilience Questionnaire” for Iranian Families. Int. J. Behav. Sci..

[38] Li X., Li H. (2021). Reliability and Validity of the Chinese Version of the Revised Walsh Family Resilience Questionnaire. Ann. Palliat. Med..

[39] Sabah A., Al-Shujairi O.K.R., Boumediene S. (2021). The Arabic Version of the Walsh Family Resilience Questionnaire: Confirmatory Factor Analysis of a Family Resilience Assessment Among Algerian and Iraq Families. Int. J. Syst. Ther..

[40] Duncan J.M., Garrison M.E., Killian T.S. (2021). Measuring Family Resilience: Evaluating the Walsh Family Resilience Questionnaire. Fam. J. Alex. Va.

[41] Maulidia F.N., Kinanthi M.R., Permata A.S., Fitria N. (2017). Family Resilience Pada Keluarga Yang Memiliki Anak Dengan Spektrum Autistik-Ditinjau Dari Perspektif Ibu. Intuisi. J. Psikol. Ilm..

[42] Gumelar G., Akbar Z., Suryaratri R.D., Erchanis H., Wahyuni L.D. (2020). The Effect of Family Resilience towards Household Disaster Preparedness in Coastal Coast District of Sumur, Banten. IOP Conf. Ser. Earth Environ. Sci..

[43] Barboza J., Seedall R. (2021). Evaluating the Relationship between Family Resilience and Grief-Related Symptoms: A Preliminary Analysis. Death Stud..

[44] Brivio E., Guiddi P., Scotto L., Giudice A.V., Pettini G., Busacchio D., Didier F., Mazzocco K., Pravettoni G. (2020). Patients Living with Breast Cancer during the Coronavirus Pandemic: The Role of Family Resilience, Coping Flexibility, and Locus of Control on Affective Responses. Front. Psychol..

[45] Mawarpury M., Hafiza S. (2019). Understanding Family Resilience in Aceh. Proceedings of the 1st International Conference on Psychology.

[46] Hadfield K., Ungar M. (2018). Family Resilience: Emerging Trends in Theory and Practice. J. Fam. Soc. Work.

[47] Masten A.S., Liebkind K., Hernandez D.J. (2012). Realizing the Potential of Immigrant Youth.

[48] Cicchetti D. (2013). Annual Research Review: Resilient Functioning in Maltreated Children—Past, Present, and Future Perspectives. J. Child Psychol. Psychiatry.

[49] Tol W.A., Song S., Jordans M.J.D. (2013). Annual Research Review: Resilience and Mental Health in Children and Adolescents Living in Areas of Armed Conflict-a Systematic Review of Findings in Low- and Middle-Income Countries. J. Child Psychol. Psychiatry.

[50] Rolland J.S., Walsh F. (2006). Facilitating Family Resilience with Childhood Illness and Disability. Curr. Opin. Pediatr..

[51] Hawley D.R. (2000). Clinical Implications of Family Resilience. Am. J. Fam. Ther..

[52] Masten A.S., Monn A.R. (2015). Child and Family Resilience: A Call for Integrated Science, Practice, and Professional Training: Child and Family Resilience. Fam. Relat..

[53] Nadrowska N., Błażek M., Lewandowska-Walter A. (2017). Family Resilience—Definition of Construct and Preliminary Results of the Polish Adaptation of the Family Resilience Assessment Scale (FRAS). Curr. Issu. Pers. Psychol..

[54] Szlendak T. (2020). Socjologia Rodziny: Ewolucja, Historia, Zróżnicowanie.

[55] Lewandowska-Walter A., Błażek M. (2018). Test Relacji Rodzinnych.

